# Scalable self-assembly interfacial engineering for high-temperature dielectric energy storage

**DOI:** 10.1016/j.isci.2022.104601

**Published:** 2022-06-11

**Authors:** Chao Wu, Anna Marie LaChance, Mohamadreza Arab Baferani, Kuangyu Shen, Zongze Li, Zaili Hou, Ningzhen Wang, Yifei Wang, Luyi Sun, Yang Cao

**Affiliations:** 1 Electrical Insulation Research Center, Institute of Materials Science, University of Connecticut, Storrs, CT 06269, USA; 2 Polymer Program, Institute of Materials Science, University of Connecticut, Storrs, CT 06269, USA; 3 Department of Chemical and Biomolecular Engineering, University of Connecticut, Storrs, CT 06269, USA; 4 Department of Electrical and Computer Engineering, University of Connecticut, Storrs, CT 06269, USA

**Keywords:** Energy systems, Interface science, Materials science

## Abstract

Flexible polymer dielectrics which can function well at elevated temperatures continue to be significant in harsh condition energy storage. However, state-of-the-art high-temperature polymers traditionally designed with conjugated structures for better thermal stability have compromised bandgaps and charge injection barriers. Here, we demonstrate a self-assembled polyvinyl alcohol (PVA)/montmorillonite (MMT) coating to impede charge carriers injecting into the polyimide (PI) polymer film. The anisotropic conductivity of the 2D nanolayered coating further dissipates the energy of charges through tortuous injection pathways. With the coating, high field pre-breakdown conduction measurement and space-charge profiling of PI films reveal a clear shifting of the dominant mode of conduction from the bulk-limited hopping to Schottky-injection limited conduction. The coating thus imparts PI films with a significantly suppressed electrical conduction (∼10×), and substantially improved discharge efficiency (7×) and energy density (2.7×) at 150°C. The facile and scalable flow-induced fabrication unleash enormous applications for harsh condition electrification.

## Highlights

• A self-assembled coating is reported to impede charge injecting into dielectrics
• The bulk-limited hopping is shifted to Schottky-injection limited conduction
• The coating improved discharge efficiency (7×) and energy density (2.7×) at 150°C
• The flow-induced fabrication is facile and scalable for enormous applications

## Graphical abstract

**Figure:**
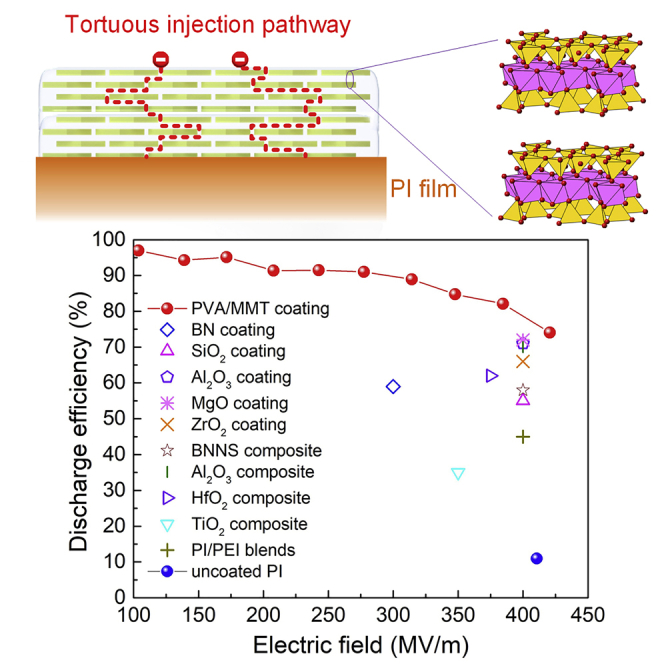

## Introduction

Dielectric thin films with ultrahigh charging-discharging rate and high voltage withstanding are critical to power converter, pulsed power, and electric propulsion applications (Mannodi-Kanakkithodi et al., 2016; Pan et al., 2019), with advantages of graceful failure mode, flexibility for roll-to-roll winding and/or conformal coating processes, and scalability for electronic device fabrication. However, conventional high-strength polymers can operate only at relatively low temperatures, e.g., of <85°C for the biaxially oriented polypropylene (BOPP) (Rabuffi and Picci, 2002; Tan et al., 2014). Such constraint brought up great challenges for emerging harsh condition electrifications in More-Electric-Aircraft (MEA), downhole oil and gas exploitations, and offshore wind power, where new wideband semiconductors (SiC, GaN) could enable system designs to offer unprecedentedly high power density and payload efficiency but would require also an operation temperature surpassing 150°C for all the components (Ho and Jow, 2012; Watson and Castro, 2015). Among the existing high-performance polymers, polyimides (PI) offer the best-balanced properties of flexibility, high mechanical strength, superior temperature, and chemical resistances, and are therefore widely used in high-end applications of aerospace and military electronics, PCBs and busbar laminates, HV isolators, and in nearly all the flexible electronics today (Ji et al., 2019). Even so, owing to their conjugated aromatic structures, PIs have intrinsically low bandgaps (Wu et al., 2020) that give rise to a drastic increase in the electrical conduction and thus limited dielectric strength at high electric fields, especially under also elevated temperatures, preventing their further use in aforementioned harsh condition electrifications (Ieda, 1984; Wu et al., 2020).

In addition to bulk limited processes of charge transport and breakdown, injection of charges from electrodes plays key roles in governing the electrical conduction of polymer dielectrics (Dissado and Fothergill, 1992; Ieda, 1984; Makasheva et al., 2016; Sessler et al., 1986; Xia and Zhang, 2004). Imperfections on the surface of polymer films stemming from complex morphological and conformational disorders of polymers introduce surface defect states, via which charges can inject into the bulk of the film over the lowered energy barrier (Coelho, 1974; Hughes, 1980; Kamal et al., 2020; Roy et al., 2005). Therefore, to explore flexible dielectric materials utilizing established high-temperature polymers, an alternative strategy is to inhibit charge injection from electrodes and thus suppress the conduction current with the interface engineering technique (Zhang et al., 2021a). Inorganics with large bandgaps were deposited on polymer film surfaces to block the charge injection, using chemical vapor deposition (CVD) and physical vapor deposition (PVD) typically requiring high-temperature processing, complicated chemical reactions, and/or high voltage plasma assistance (Azizi et al., 2017; Zhou et al., 2018). Despite these cumbersome and laborious processes, risks remain during scaling-up as essentially a single pinhole may lead to the complete failure of the system. Organic-inorganic composite materials were also demonstrated to improve the dielectric properties at elevated temperatures, while they cannot directly utilize commercially available dielectric films, which also limited the scaling-up (Ai et al., 2020; Yu et al., 2022). It is desirable to develop facile and scalable coating techniques with high throughput to further expand surface engineering techniques to actual applications.

We demonstrated a facile flow-induced self-assembly coating approach for re-surface engineering (Zhang et al., 2021a). Compared to chemical vapor or physical deposition of inorganics, solution-based coating is economically more scalable and higher throughput (Zhu et al., 2016). Specifically, we introduced a polyvinyl alcohol (PVA)/montmorillonite (MMT) solution-based self-assembly coating with hundreds of highly ordered organic/inorganic alternating layers on Kapton Polyimide (PI) films. The 2D nanosheets are engineered to form a high-quality defect-tolerant coating and the large band gap of MMT is employed to help to suppress the charge injection (Zhang et al., 2021b; Zhu et al., 2016). In this work, we focus on the high-temperature dielectric behavior of PI with PVA/MMT self-assembly coating.

## Results and discussion

**Figure 1:**
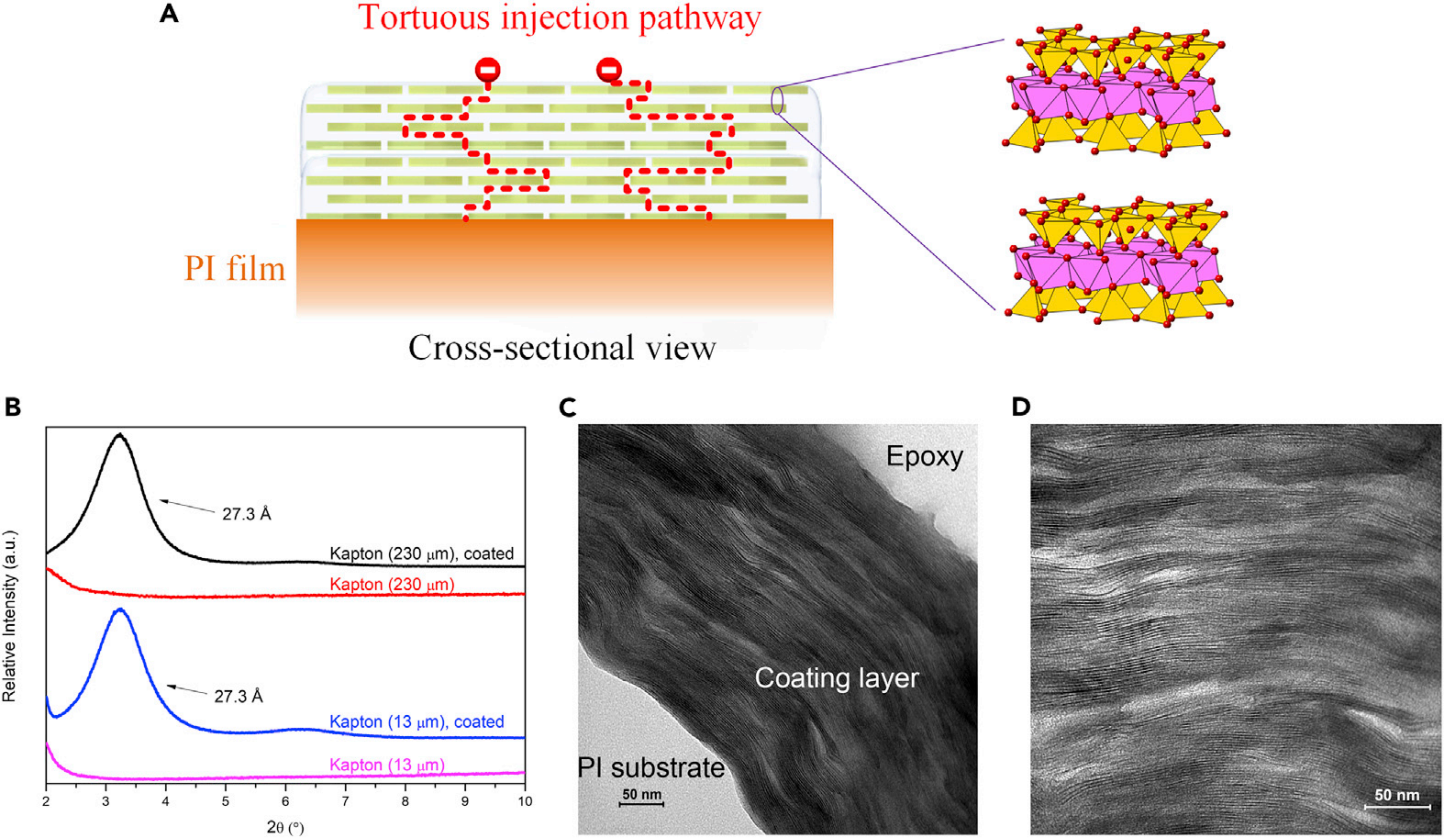
Materials and characterization (A) Schematic of the coating and tortuous injection pathways of electrons. (B) XRD patterns of the coated PI films. (C and D) TEM images of the cross-section of the nanocoating at low (C) and high (D) magnifications.

As schematically shown in Figure 1A, the multilayered, highly ordered structure of the coating is expected to not only impede charge injection via the revived Schottky barrier but also further dissipate the hot electron energies through tortuous layered injection pathways. PI film of 230 μm is employed for the study of space charge distribution by using the pulsed electro-acoustic (PEA) technique (thick films are needed owing to the limited spatial resolutions of PEA) (Tefferi et al., 2019). All other investigations are based on PI films with a thickness of 13 μm. The flexible and transparent PI films with a thickness of 13 μm with or without coating are shown in Figure S1. The high transparency of the coated PI film indicates that the MMT nanosheets were well-assembled with a high degree of orientation, thus minimizing the light scattering. The XRD patterns of the coated PI films are shown in Figure 1B. The intensive basal diffraction peaks again suggest that a well-ordered layered structure has been formed on the two PI films. As expected, the substrate thickness barely affects the overall coating structure. The interlayer distance of the assembled MMT nanosheets was ca. 2.73 nm on each substrate. TEM images of the cross-section of the nanocoating (on 13 μm PI film) at different magnifications are shown in Figures 1C and 1D. A well-oriented, layered structure of nanosheets is observed, which is in good agreement with the XRD characterization. Considering the coated samples are designed for high-temperature applications, the nanocoatings were also crosslinked to achieve high stability. The evidence of crosslinking is shown in the FTIR spectra in Figure S2.

**Figure 2:**
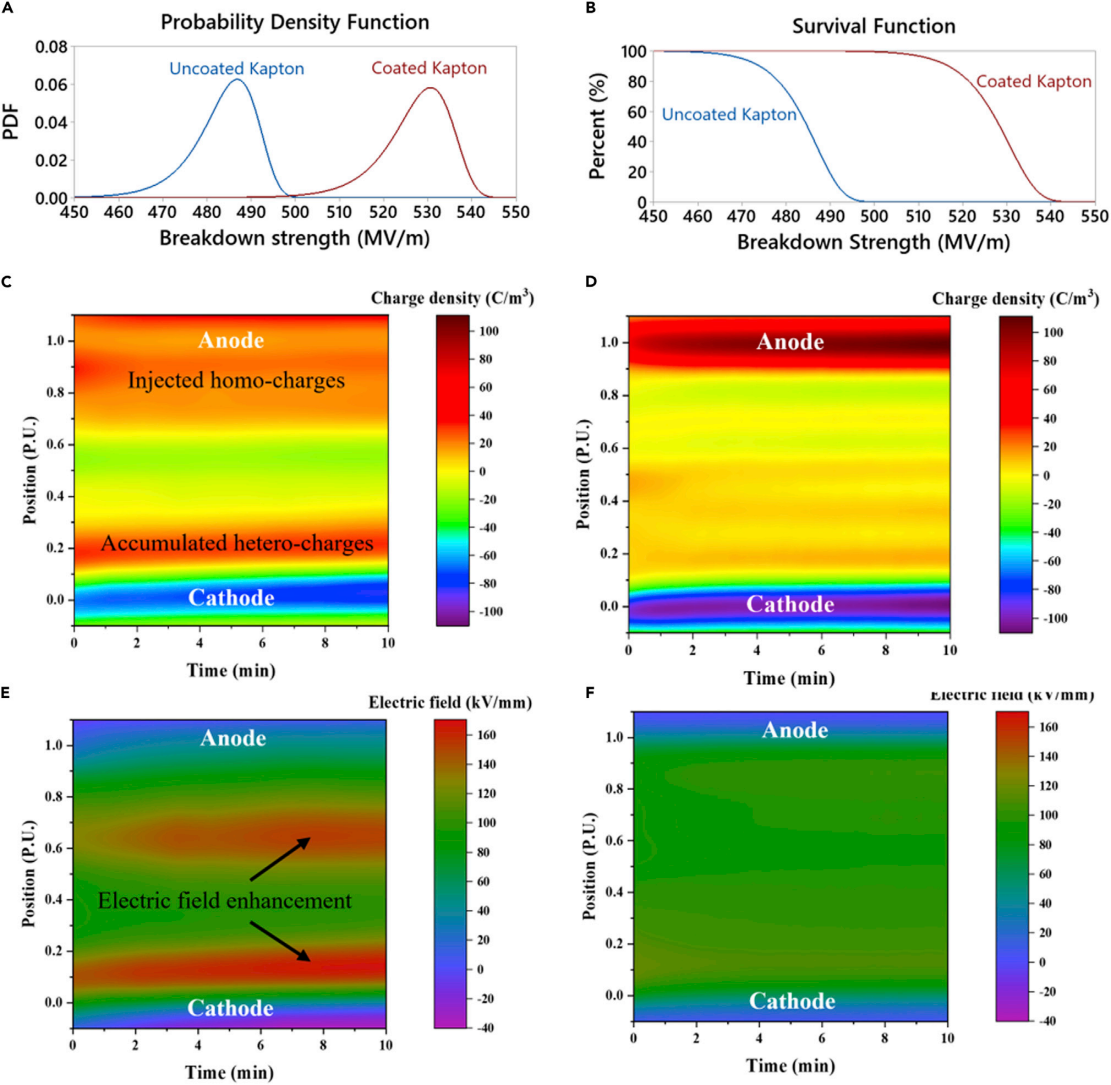
Charge injection and breakdown strength (A and B) Characteristics of the breakdown strength from Weibull distribution. (C and D) Injected charge densities in (C) uncoated PI and (D) coated PI. (E and F) Electric field distribution in the (E) uncoated PI and (F) coated PI film after charge injection.

Breakdown strength is the foremost parameter for harsh-condition electrifications. To demonstrate the defect tolerance capability of the nanosheets coating, the characterization of breakdown strength was performed based on 50 points of measurements. The probability function and survival function of Weibull fittings indicate higher breakdown strength of the coated PI films (Figures 2A, 2B, S3, and S4). From the survival function fitting, it can be clearly observed that even the breakdown strength minimum of the coated film is higher than the breakdown strength maximum of the uncoated film. The characteristic breakdown strength evaluated by the Weibull model at the 63.2% (1-1/e) failure probability is improved from 487 MV/m to 530 MV/m with the coating. The shape factor of the Weibull breakdown strength for the coated film is as high as 84, indicating the homogeneity of the coating. The high breakdown strength of 50 data points, each measured over a large active area of 1 cm by 2 cm, also demonstrated the superior defect tolerance capability of the coating. The morphology of the breakdown regions for the uncoated and coated PI films was shown in Figure S5 to exhibit the damage patterns following a dielectric breakdown. Extensive radial traces are apparent for the coated films, which result from the tortuous charge-spreading pathways branching along and between the highly aligned inorganic MMT nanosheets. To experimentally demonstrate the blocking effect of the coating, we investigated the charge distribution across the thickness of PI films under 100 kV/mm for 10 min by using the PEA method as illustrated in Figures 2C and 2D (Tefferi et al., 2019). The position of interest across the thickness is normalized to the total thickness of the film (230 μm) and presented per unit (P.U.) in which zero and one correspond to the positions of cathode (negative electrode) and anode (positive electrode), respectively. With the layered nanosheets coating, the PI film shows significant suppression for both the homo-polar and the hetero-polar charges that would otherwise present in the uncoated PI owing to the charge injection next to the anode and the accumulation of the migrated charges under the electric field close to the cathode. In addition, the electric field distribution related to the charge density profile could be calculated using the Gauss’s law as presented by (1)

(Equation 1) $$E\left(x\right)=\frac{1}{\varepsilon_{0}\varepsilon_{r}}\int_{0}^{x}\rho \left(x\right)dx$$

where ρ(x) is the charge density, *E(x)* is the electric field, $\varepsilon_{0\;}$ is the vacuum permittivity, and $\varepsilon_{r}$ is the relative permittivity of the material. The electric field distribution after charge injection is illustrated in Figures 2E and 2F. Owing to the injection and accumulation of charge across the specimen especially adjacent to the cathode, the electric field in the bulk of the PI film is highly distorted by the local field induced by the space charges; contrarily, in the coated PI film, a much uniform electric field distribution is presented, attributed to less charge injection through the coating. For the uncoated PI, the maximum local field reaches up to 168 kV/mm (a +68% enhancement) while it is only 118 kV/mm (+18%) in the coated PI film (Figure S6). The largely suppressed conduction owing to less charge injection and more uniform electric field distribution across the specimen is beneficial to the higher breakdown strength and higher operational field.

**Figure 3:**
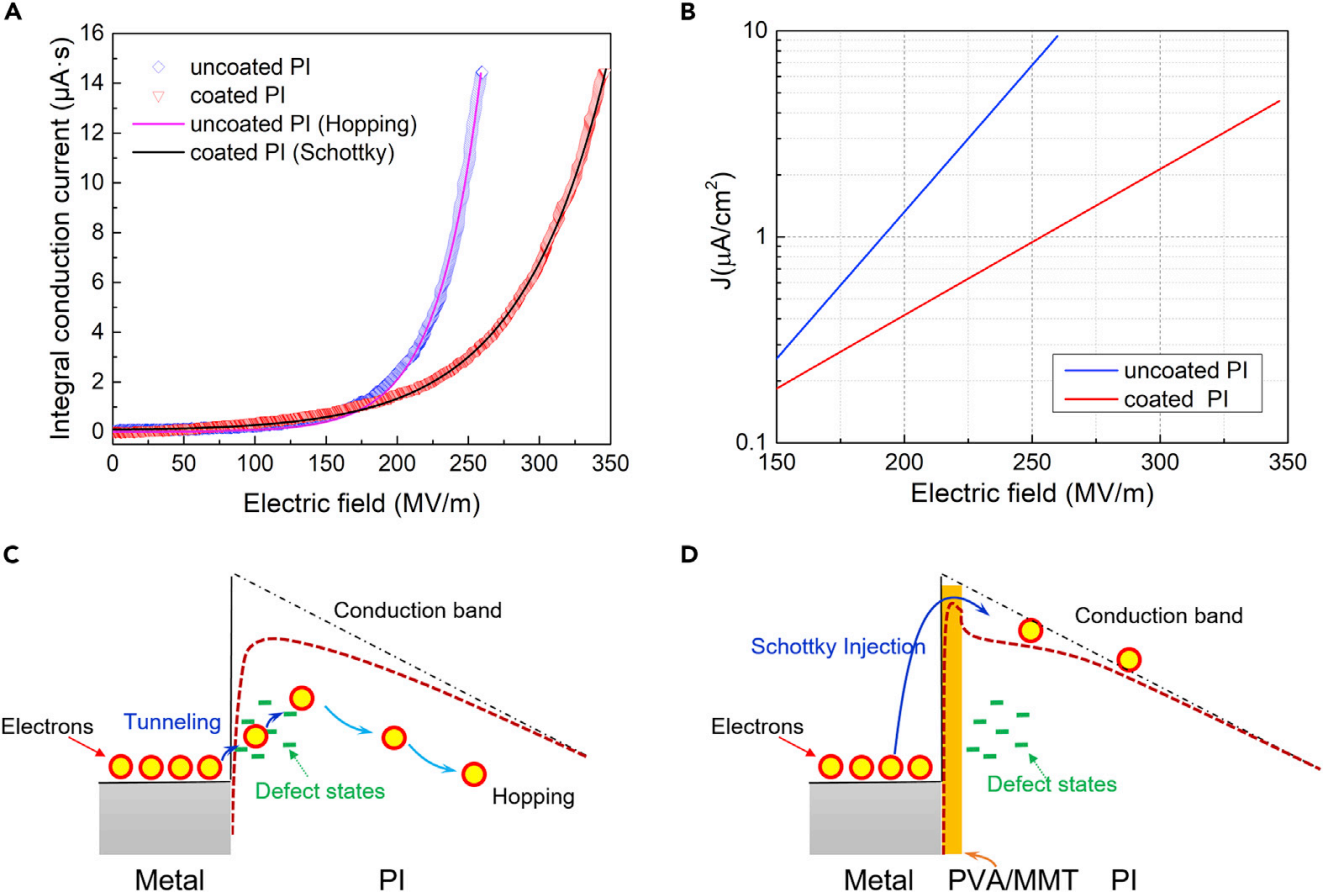
Electrical conduction (A) Integral conduction current at 150°C. (B) Conduction current density at 150°C. (C) Schematic of bulk limited conduction for uncoated PI. (D) Schematic of Schottky injection interface limited conduction for PVA/MMT coated PI.

For capacitive energy storage, conduction loss is the major source of energy dissipation under high electric fields and elevated temperatures. The high field conduction was experimentally investigated using a designed system that can dynamically cancel the capacitive component of the current. The remaining signal output represents the time-integrated conduction current across the sample, with accuracy for the small resistive current down to 10 ppm (Li et al., 2016). The integral conduction current (*ICC*) and conduction current density under 150°C are shown as a function of the electric field (Figures 3A and 3B). The conduction model taking into account the Schottky effect is used to reveal the impact of the coating on charge injection, as expressed in Equations (2) and (3) (Sessler et al., 1986).

(Equation 2) $$J=AT^{2}\;\exp\left[-\left(\phi -\beta E^{1/2}\right)/kT\right]$$

with

(Equation 3) $$\beta ={\left(e^{3}/4\pi \varepsilon_{0}\varepsilon_{r}\right)}^{1/2}$$

where *J* is the conduction current, *A* is a constant, *T* is the temperature in Kelvin, *E* is the electric field, *ϕ* is the injection barrier, *k* is the Boltzmann constant, *e* is the electronic charge, *ε*_0_ is the vacuum permittivity, and *ε*_r_ is the relative permittivity. Significant parameters can be obtained by combining the integral relationship between ICC and *J* in Equation (4).

(Equation 4) ICC=∫Jdt

The calculated *ε*_r_ for uncoated PI is 1.2, which is much smaller than the measured *ε*_r_ (3-3.2, Figure S7), indicating that the charge transport behavior for uncoated PI deviates significantly from the interface limited conduction model (Figure 3C). However, the calculated *ε*_r_ for coated PI is 3.6, slightly higher than the measured *ε*_r_ (3-3.25), suggesting a plausible Schottky barrier (interface) limited charge transport (Figure 3D). For uncoated PI, defect formation through the long chain disorder of polymers in the interface region leads to charge injection over a much smaller energy barrier compared to the theoretic Schottky barrier. As a result, the charge transport is controlled by the bulk of the film rather than by the interface. The well-aligned, highly ordered structure of the PVA/MMT coating revived the Schottky barrier of PI, restoring the interface limited conduction. The conduction current of uncoated PI is, therefore, analyzed using the hopping conduction model (experimental section). Attributed to the revived Schottky barrier, the conduction current was nearly one order of magnitude lower with the coating, on a side-by-side comparison with uncoated PI (Figure 3B).

**Figure 4:**
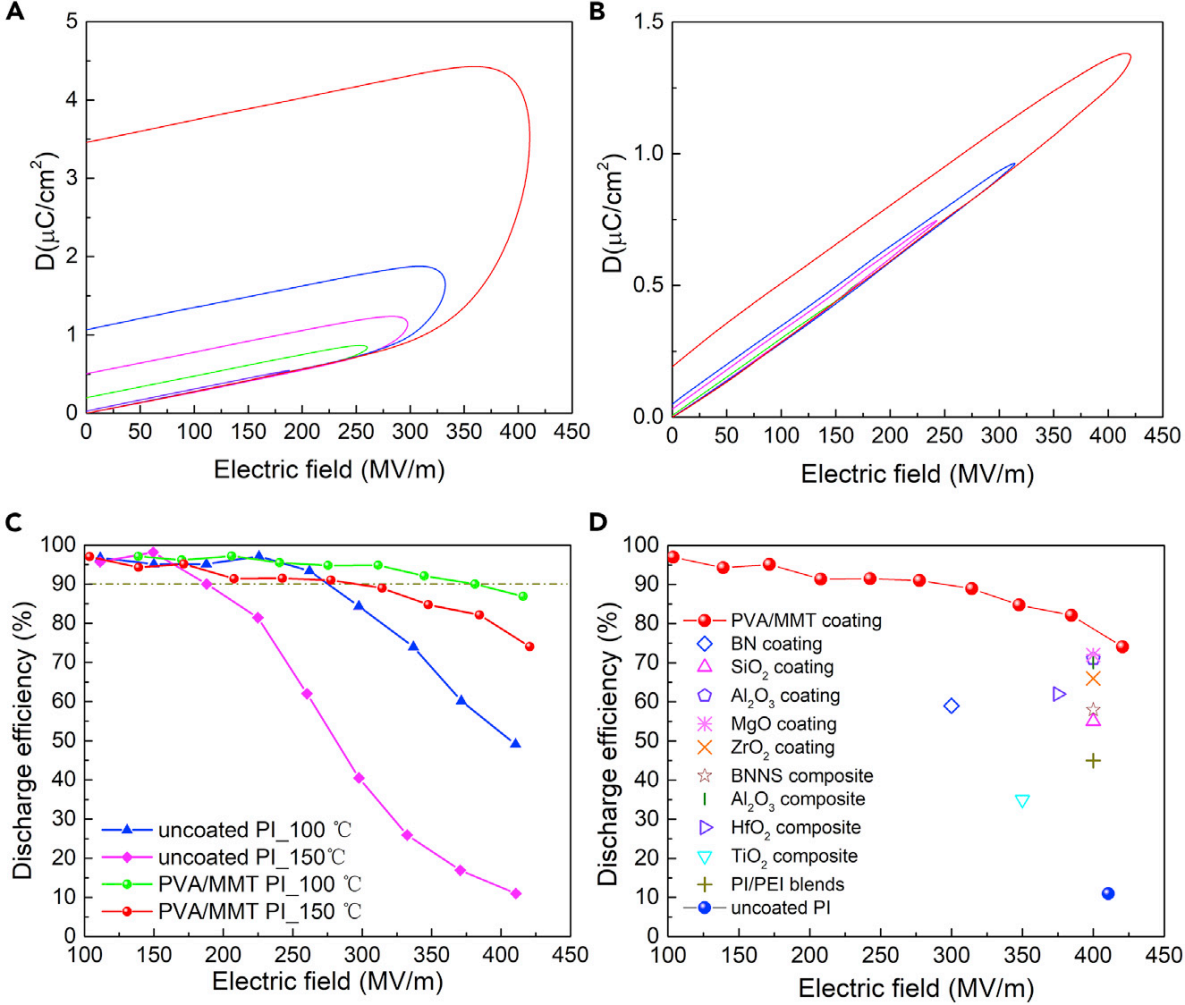
Energy storage performance (A and B) DE loops of (A) the uncoated PI film and (B) the coated PI film at 150°C. (C) Discharge efficiency under elevated temperatures as a function of the electric field at 100°C and 150°C. (D) Discharge efficiency of the PVA/MMT coated PI relative to reported PI-based dielectrics at 150°C.

The high electric field energy storage performance was characterized via displacement-electric field (DE) loops. The area inside the loops represented energy dissipation. At the same electric field, PI films with the coating exhibited much narrower DE loops relative to the uncoated films (Figures 4A and 4B), especially at 150°C, indicating the suppression of energy loss by the coating. Figure 4C summarized the charge-discharge efficiency (*η*), which is the ratio of discharged energy density and the total input energy density, of PI films under 100°C and 150°C as a function of the electric field. As the Joule heating produced by the conduction current can lead to thermal breakdown of dielectric materials at electric fields far below their intrinsic breakdown strength, dielectrics with low charge-discharge efficiency are not suited for capacitors applications. Therefore, we selected 90% as a fixed efficiency to make comparisons among coated and uncoated films under different temperatures. With the electric field increasing, the discharged energy density increased quadratically (*U*_e_ = 1/2*ε*_0_*ε*_r_*E*^2^, where *U*_e_ is the discharged energy density) while the charge-discharge efficiency decreased owing to the exponentially increased conduction loss. The electric field correlated with the efficiency (*η*) of 90% is marked as *E*_90_. Under 100°C, *E*_90_ is 280 MV/m for the uncoated film, which is improved to 380 MV/m for the coated film. Under 150°C, *E*_90_ is 188 MV/m and 314 MV/m for the uncoated and coated films, respectively. With the coating, *E*_90_ was increased by 36% under 100°C and 67% under 150°C. Contributions of the coating could also be evaluated by the temperature dependence of *E*_90_ from another perspective. From 100°C to 150°C, a more intense charge injection gave rise to higher conduction loss. *E*_90_ was decreased by 33% for the uncoated PI film but only 17% for the coated film. The less temperature dependence of *E*_90_ demonstrated again the blocking effect of the coating in charge injection and regulation of the conduction current at elevated temperatures. The discharged energy densities with an efficiency above 90% were shown in Figure S8, which was improved from 0.48 J/cm^3^ to 1.3 J/cm^3^ (2.7×) at 150°C. The charge-discharge efficiency at 150°C for the PVA/MMT coated PI outperforms the best-reported PI composites or PI with coatings from a side-by-side comparison (Figures 4D and Table S1) (Ai et al., 2020; Azizi et al., 2017; Dong et al., 2021; Zhang et al., 2021b; Zhou et al., 2018). Although PI with one extra Al_2_O_3_ layer inside the PI film (Al_2_O_3_-PI-Al_2_O_3_-PI-Al_2_O_3_) shows slightly higher efficiency at ∼400 MV/m, the complicated composite architecture is not applicable for commercially available polymer films (Dong et al., 2021). Instead, this facile, highly scalable assembly of the MMT nanosheets coating can be readily applied to PI and other polymer films for mass production.

MMT nanosheets are covered by PVA chains when they are mixed in an aqueous suspension (Ding et al., 2017). Thus, there will be virtually no direct bonding between MMT and PI. Instead, a thin layer of PVA is bonded with PI. The PI surface was corona discharge treated and thus it became hydrophilic, generating a strong interface with PVA. As a result, the coating layer is very difficult to be peeled off the substrate (damaged during the peeling process), indicating the strong bonding between the coating and the PI substrate. This method is universal for various types of polymer substrates and 2D nanosheets. We have coated on many other different polymer substrates including polylactic acid (PLA), polyethylene terephthalate (PET), polypropylene (PP), high-density polyethylene (HDPE), low-density polyethylene (LDPE) (Ding et al., 2017; Zhang et al., 2021b), polystyrene (PS), polyvinylidene fluoride (PVDF), paper (Chavez et al., 2021), cardboard (Williams et al., 2021), and cotton fabrics (Zhang et al., 2017, 2018, 2020, 2021c). We have also used many different 2D nanosheets including MMT (Ding et al., 2017; Zhang et al., 2021c), Laponite (Liu et al., 2021), graphene oxide (Liu, 2018), layered double hydroxide (Yu et al., 2016), and α-zirconium phosphate (Liu, 2018).

### Conclusions

In summary, we demonstrated a flexible high-temperature dielectric film by incorporating a 2D nanosheets coating on both sides of the commercially available polyimide films. A flow-assisted approach was developed to assemble the laminated single-layer nanosheets on the surface of polymer dielectric films. High field conduction studies show a clear shifting of conduction modes from the bulk-limited hopping conduction in uncoated PI to electrode-limited Schottky injection in PI with the nanoclay coating. The great suppression of conduction loss gave rise to significantly improved charge-discharge efficiency under high electric fields and largely improved overall breakdown strength and energy storage densities, especially at elevated temperatures. The facile and scalable fabrication, and more importantly, the defect tolerance from the well-aligned laminated structure reveals its tremendous potential in resurrecting commodity high-temperature polymer dielectrics like Kapton PI for extensive uses in the rapidly expanding, large scale harsh-condition electrifications.

### Limitations of the study

In the follow-up research, it is expected to further investigate the long-term stability of the coated PI films and integrated capacitors under concurrent elevated temperature and high electric field.

## STAR★Methods

### Key resources table

**Table:** 

REAGENT or RESOURCE	SOURCE	IDENTIFIER
**Chemicals, peptides, and recombinant proteins**		

Polyimide (PI)	American Durafilm	50HN (.0005") Kapton® Film x 25"
polyvinyl alcohol (PVA)	Kuraray	Mowiol® 8-88
montmorillonite (MMT)	Minerals Technologies Inc.	PGN

### Resource availability

#### Lead contact

Further information and requests for resources and reagents should be directed to and will be fulfilled by the lead contact, Luyi Sun (luyi.sun@uconn.edu) and Yang Cao (yang.cao@uconn.edu).

#### Materials availability

This study did not generate new unique reagents.

### Experimental model and subject details

This work did not need any unique experimental model.

### Method details

#### Materials and characterizations

To fabricate the self-assembled coating, a dispersion of well-exfoliated montmorillonite (MMT) nanosheets and polyvinyl alcohol (PVA) (0.75 wt. % MMT + 0.75 wt. % PVA + 98.5 wt. % water) was prepared in advance. The low-viscosity dispersion helped to generate a quick flow and a thin liquid layer, both of which are favorable for achieving a high level of orientation of MMT nanosheets. The corona discharge treated PI films with two thicknesses, 13 and 230 μm, were dip-coated with the above-mentioned aqueous dispersion and then vertically hung in an oven to be dried and crosslinked at 60°C. Thickness measurements of the nanocoatings were performed on a Semiconsoft MProbe Thin Film Measurement System (Southborough, MA). For the coated Kapton substrate, at least 10 thickness measurements were performed. The average results with standard deviations are reported. The resulting coating layer was measured to be 347 ± 15 nm. This thickness results in a complete coverage of the substrate material with a defect-free charge-injection capability while maintaining the flexibility of the PI film, contributing to an optimized breakdown strength. The thickness of the self-assembly coating can also be adjusted by changing the cycle of dip coating.

The X-ray diffraction (XRD) patterns were recorded on a Bruker D2 diffractometer using a graphite monochromator with Cu K α radiation. Cross-sectional TEM images of the nanocoating structure were captured using a Tecnai T-12 TEM. The coated PI film (13 μm in thickness) was embedded into epoxy and microtomed into thin slices (thickness ca. 100 nm) on a Reichert-Jung Ultracut E ultramicrotome. The cross-sections were placed onto a 400-mesh copper grid with a carbon supporting film for imaging at an accelerating voltage of 100 kV.

FTIR spectra were recorded on a Nicolet Magna-IR 560 spectrophotometer. Freestanding thin films were delaminated from the substrates for FTIR characterization. To verify the crosslinking of the nanocoatings on the PI films, the FTIR spectrum of the delaminated nanocoating was collected (Figure S1). For comparison, the spectrum for the crosslinked nanocoating (PVA/MMT-C) is compared with the spectra for MMT, PVA, crosslinked PVA (PVA-C), and non-crosslinked nanocoating (PVA/MMT-N). The additional peak at ca. 1106 cm^−1^ for the crosslinked coating is an indicator of the formation of Si-O-C bonds from the reaction between the Si-OH groups in MMT and the C-OH groups in GA. Upon crosslinking, there is also a reduction in intensity in O-H stretching (ca. 3370 cm^−1^) and C-OH stretching (ca. 1060 cm^−1^).

#### Electrical measurement

Breakdown-strength measurements were performed at room temperature under a direct-current voltage ramp of 300 V/s, and the active testing area was 1 × 2 cm^2^. The electrodes on the test sample films are evaporated aluminum (Al) metalized BOPP films. A thicker Kapton PI film (120 μm) with a window of 1 × 2 cm^2^ was inserted between the sample under investigation and the high voltage metalized film electrode to control the active electrode area (Figure S3). The high voltage was generated by a Stanford PS375 high-voltage power supply and controlled by a ramp-signal generator.

The space charge distribution was measured using Pulsed Electro Acoustic (PEA) method(Arab Baferani et al., 2021; Tefferi et al., 2019). The acoustic pulse was obtained by the application of the voltage impulses with 1–2 ns rise time and a peak voltage of 350V. For PEA measurement, the specimens were coated on both sides with 60/40 weigh% Au/Pd sputter coating with a diameter of 1 cm.

The conduction at the high field was measured with a specially designed capacitive cancellation measurement system (Li et al., 2016). The system uses a small sinusoidal modulation signal superimposed on the voltage ramp to track the capacitive current under transient condition. Using the negative feedback loop formed with a dual-phase lock-in amplifier, the capacitive current can be actively canceled via dynamic gain control throughout the measurement. The hopping conduction model in Equation (5) described the electric field dependence of the conduction current, where *J*_0_ is proportional to the density of injected charge carriers, and *λ* is the hopping distance. The hopping distance was 2.39 nm for the uncoated PI film.

(Equation 5) $$J=J_{0}\;\sinh\left(\frac{eE\lambda }{2\text{k}T}\right)$$

The dielectric spectroscopy measurement was conducted using a Solartron SI 1260 frequency response analyzer with a Solartron 1296 dielectric interface. The sample in a test cell was put in an oven with a Delta Design 9015 temperature controller, which can control the temperature fluctuation within ±0.5°C in the whole measurement. The measurement was carried out at temperature starting from 30°C to 150°C. Before each measurement, a 30-min stabilization at a set temperature was used to guarantee the sample was in a uniform steady isothermal state.

DE loop was employed using a modified Sawyer-Tower polarization loop tester with a unipolar positive half sinusoidal wave of 100 Hz. The measurement system consisted of a Trek Model 10/40 10 kV high voltage amplifier and an OPA541 operational amplifier-based current-to-voltage converter. Gold/Palladium electrodes are coated on both sides of the film with a diameter of 3 mm using the sputter coating method to ensure a good contact between electrodes and the film.

### Quantification and statistical analysis

Our study doesn’t include quantification or statistical analysis.

## Data Availability

All data are available in the paper and in supplemental information, and/or from the corresponding authors upon reasonable request. This paper does not report original code.
