# Supplementary material for: Scalable self-assembly interfacial engineering for high-temperature dielectric energy storage
Source: iScience. 2022 Jun 11;25(7):104601. doi: 10.1016/j.isci.2022.104601 (PMC9250013; doi:10.1016/j.isci.2022.104601)
Supplement: Document S1. Figures S1–S8 and Table S1 [file mmc1.pdf]

iScience, Volume *25*

Supplemental information

Scalable self-assembly interfacial engineering for high-temperature dielectric energy storage

Chao Wu, Anna Marie LaChance, Mohamadreza Arab Baferani, Kuangyu Shen, Zongze Li, Zaili Hou, Ningzhen Wang, Yifei Wang, Luyi Sun, and Yang Cao

Supplementary Information

Scalable self-assembly interfacial engineering for high temperature dielectric energy storage

Chao Wu[1], Anna Marie LaChance[2,3], Mohamadreza Arab Baferani[1,4], Kuangyu Shen[2,3], Zongze Li[1,4], Zaili Hou[2,3], Ningzhen Wang[1], Yifei Wang[1], Luyi Sun[2,3]*, Yang Cao[1,4]*

[1]Electrical Insulation Research Center, Institute of Materials Science, University of Connecticut, Storrs, CT 06269, United States

[2]Polymer Program, Institute of Materials Science, University of Connecticut, Storrs, CT 06269, United States

[3]Department of Chemical and Biomolecular Engineering, University of Connecticut, Storrs, CT 06269, United States

[4]Department of Electrical and Computer Engineering, University of Connecticut, Storrs, CT 06269, United States

a. b.

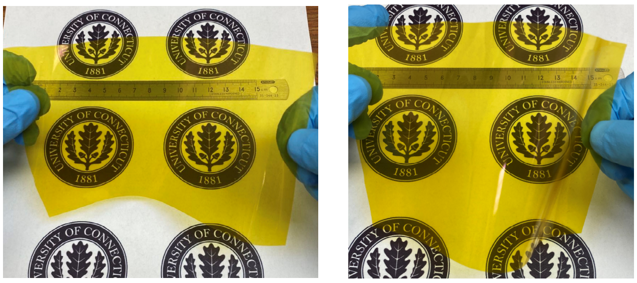

Figure S1. Photographs of (a) uncoated and (b) coated PI films.

Related to Figure 1.

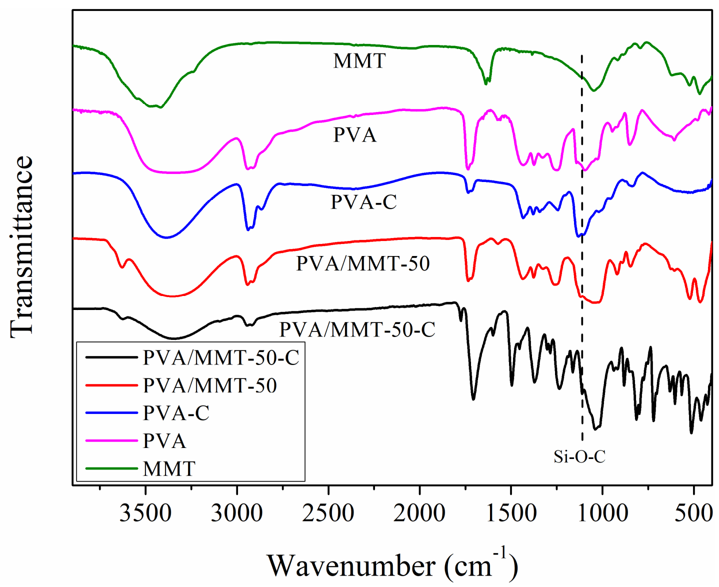

Figure S2. FTIR spectra of the nanocoatings. Related to STAR Methods.

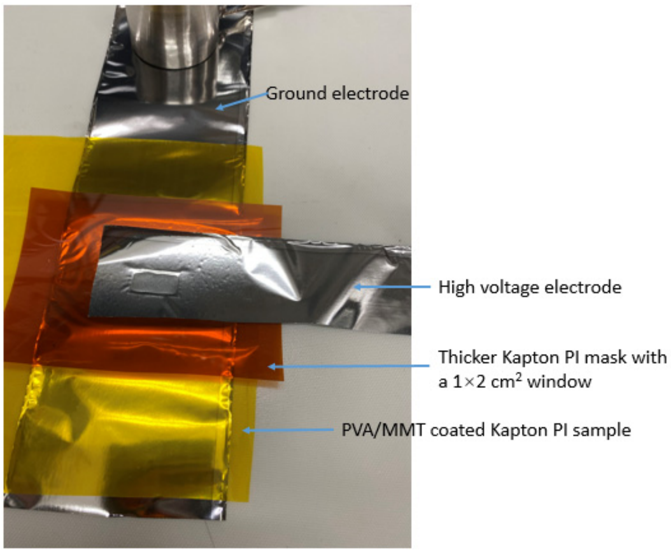

Figure S3. Setup of the breakdown experiments. Related to Figure 2.

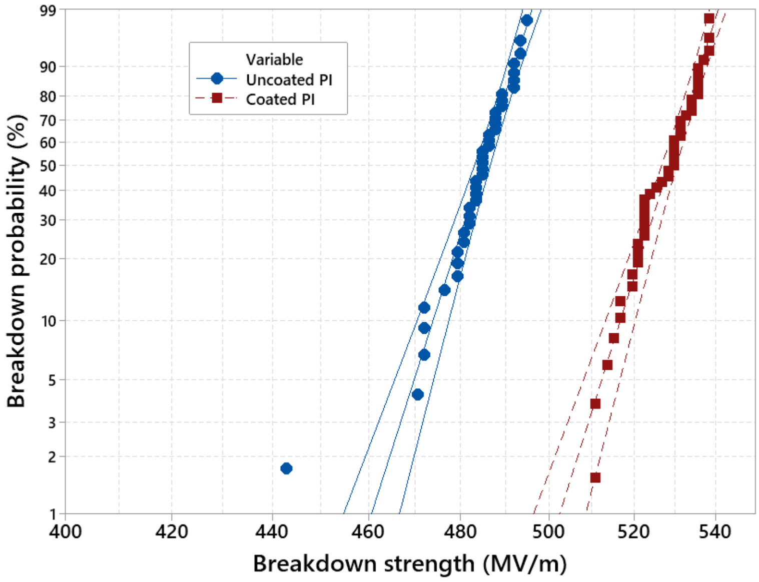

Figure S4. Weibull distribution of the breakdown strength for uncoated and coated PI. Related to Figure 2.

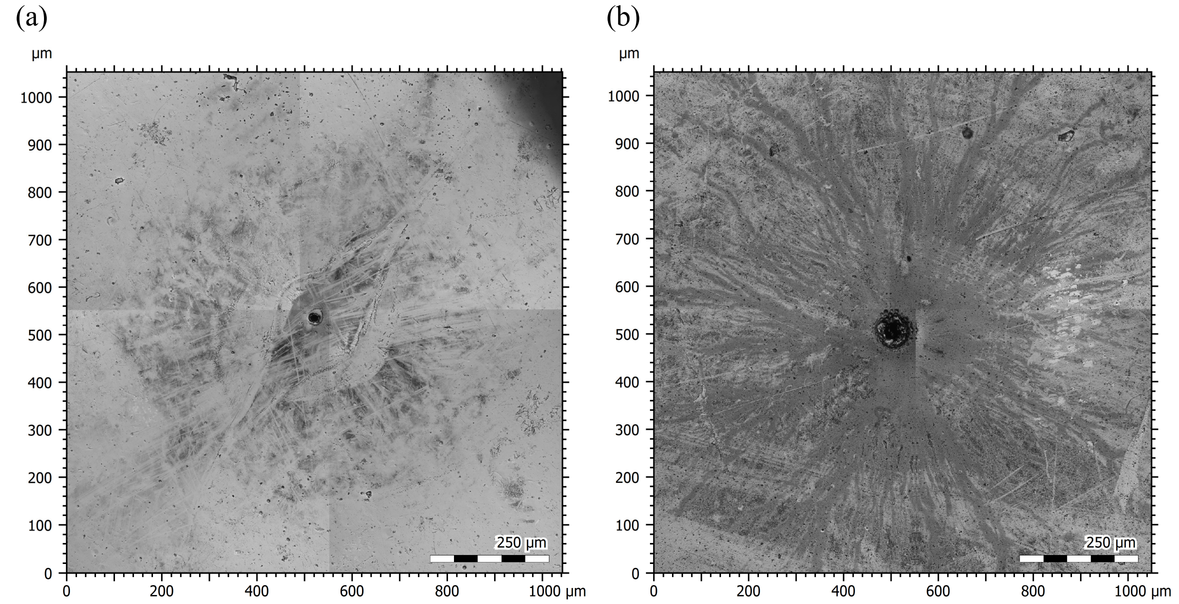

Figure S5. Morphology of the breakdown regions for the uncoated (a) and coated (b) PI film. Related to Figure 2.

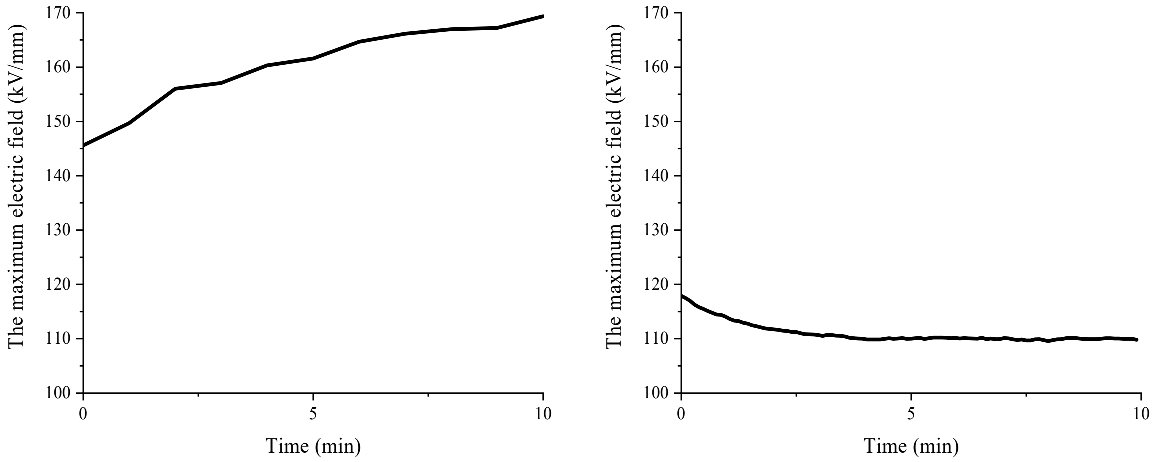

Figure S6. Maximum electric field of (a) uncoated and (b) coated PI film after charge injection under 100 MV/m. Related to Figure 2.

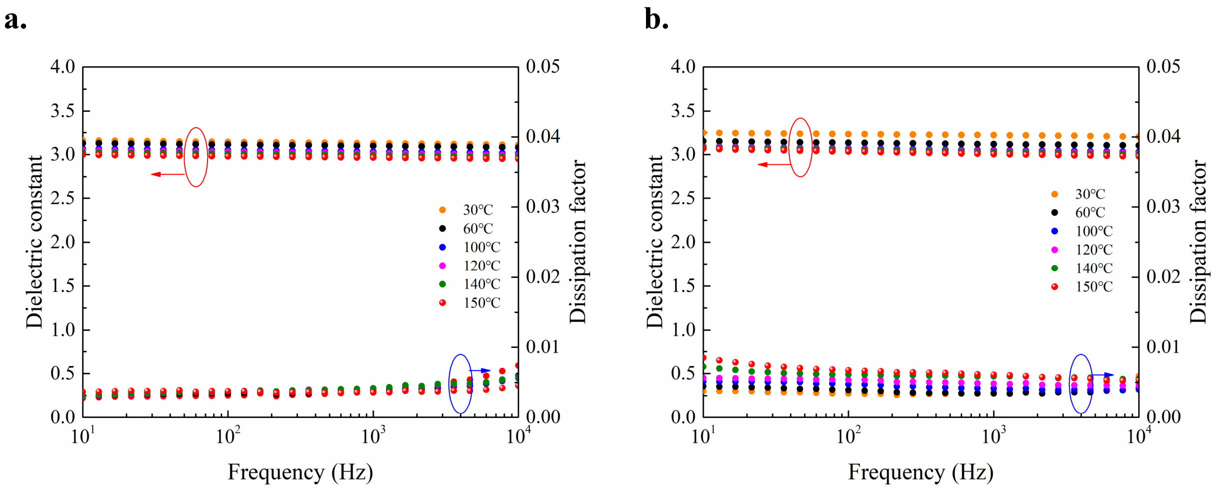

Figure S7. Electric polarization. a–b) Dielectric constant and dissipation factor for (a) the uncoated PI film and (b) the coated film. Related to Figure 3.

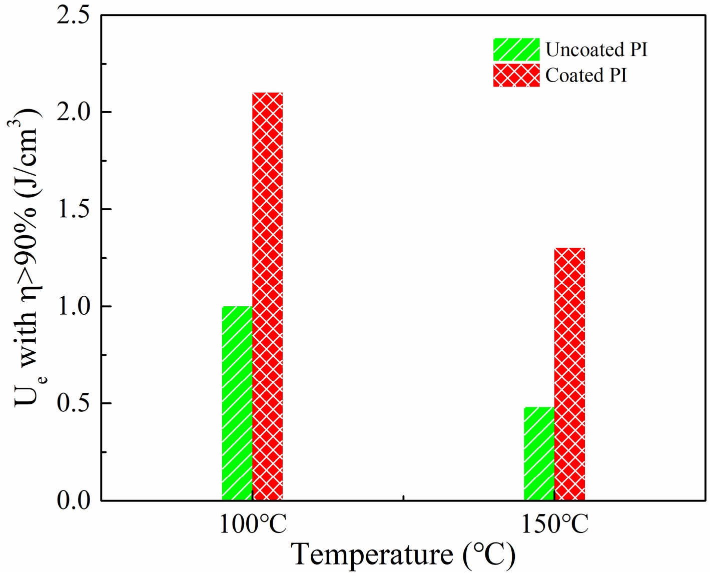

Figure S8. Comparison of discharged energy density (U_e) with the efficiency η>90%. Related to Figure 4.

Table S1. Discharge efficiency of the PVA/MMT coated PI relative to reported PI based dielectrics at 150 °C. Related to Figure 4.

**PI based dielectrics**	**Electric field (MV/m)**	**Efficiency (%)**	**Reference**
PVA/MMT coating	300<br>350<br>400	90<br>85<br>79	This work
Uncoated PI	300<br>350<br>400	79<br>22<br>12	This work
BN coating	300	59	(Azizi et al., 2017)
SiO_2 coating	400	55	(Zhou et al., 2018)
Al_2O_3 coating	400	71	(Dong et al., 2021)
MgO coating	400	72	(Dong et al., 2021)
ZrO_2 coating	400	66	(Dong et al., 2021)
BNNS composite	400	58	(Ai et al., 2020)
Al_2O_3 composite	400	61	(Ai et al., 2020)
HfO_2 composite	375	62	(Ai et al., 2020)
TiO_2 composite	350	35	(Ai et al., 2020)
PI/PEI blends	400	45	(Zhang et al., 2021)
